# 12-month survival in nonagenarians inside the Mugello study: on the way to live a century

**DOI:** 10.1186/s12877-022-02908-9

**Published:** 2022-03-12

**Authors:** Silvia Pancani, Gemma Lombardi, Francesco Sofi, Anna Maria Gori, Roberta Boni, Chiara Castagnoli, Anita Paperini, Guido Pasquini, Federica Vannetti, Raffaello Molino Lova, Claudio Macchi, Francesca Cecchi

**Affiliations:** 1 IRCCS Fondazione Don Carlo Gnocchi, Via di Scandicci, 269, 50143 Florence, Italy; 2 Department of Experimental and Clinical Medicine, University of Florence, Florence, Italy; 3 Atherothrombotic Unit, Careggi University Hospital, Florence, Italy

**Keywords:** Longevity, Physical activity, Cognitive impairments, Functional status, Nonagenarians

## Abstract

**Background:**

Life expectancy has increased over the last century and a growing number of people is reaching age 90 years and over. However, data on nonagenarians’ health trends are scarce due to difficulties in investigating this specific population. This study aims to identify risk factors for one-year mortality in nonagenarians using data collected within the “Mugello Study”.

**Methods:**

Complete information on sociodemographic data, cognitive and functional status, lifestyle, medical history, and drug use was collected from 433 nonagenarians, as well as information about survival after 1 year from the interview.

**Results:**

The sample included 314 women (72.5%) and 119 men (27.5%) with a median age of 92 years (range 90-99 years). The mortality rate was 20.3% (88 deaths). After adjustment for age and sex, a significantly higher risk of dying within 12 months was observed in individuals with more severe cognitive impairment (HR = 5.011, *p* < 0.001), more severe disability in basic activities of daily living (HR = 4.193, *p* < 0.001), sedentary lifestyle (HR = 3.367, *p* < 0.001), higher number of drugs assumed (HR = 1.118, *p* = 0.031), and kidney dysfunction (HR = 2.609, *p* = 0.004). When all the variables were included in the analysis, only older age (HR = 1.079, *p* = 0.048), lower cognitive function (HR = 2.859, *p* = 0.015), sedentary lifestyle (HR = 2.030, *p* = 0.026), and kidney dysfunction (HR = 2.322, *p* = 0.018) remained significantly associated with reduced survival.

**Conclusions:**

Data from the Mugello study support the hypothesis that survival at 12 months in nonagenarians is not a stochastic process and that older age, reduced cognitive function, sedentary lifestyle, and the presence of kidney dysfunction are associated with mortality.

**Supplementary Information:**

The online version contains supplementary material available at 10.1186/s12877-022-02908-9.

## Introduction

During the last century, the number of subjects reaching advanced age, often referred to as “oldest old”, has consistently increased in developed countries [1] and life expectancy is projected to lengthening even further in the next decades. In the European countries, the number of people aged 85 years or more (13.8 million in 2018) is expected to reach 31.8 million by 2050, while centenarians (nearly 106,000 in 2018) are expected to be almost half a million by 2050 [2].

As a consequence, the rapidly growing proportion of the aging population has become one of the most important demographic phenomena of modern society. Previous studies have aimed to identify the factors associated with reaching 90 years of age. Findings from those works suggest that risk factors such as smoking [3, 4], diabetes [3], hypertension [3–5], high levels of total cholesterol [4], and sedentary life [4, 6] as well as a high BMI [3, 5, 6], a low income [4], and being unmarried [4] decrease the likelihood of reaching 90 years of age. However, reaching the age of 90 years does not necessarily coincide with the end of life; as life expectancy is increasing, a significant number of people at this age are likely to live for several years more [7]. For this reason, some studies have investigated whether known or suspected risk factors for mortality in older adults are also important in the nonagenarians, whether these predictors lose their importance, or their effect is reversed. Martel and colleagues [8] investigated the predictors of 5 years survival in a cohort of 124 hospitalized nonagenarians. They found Charlson index and Barthel index to be independently related to long-term survival. Formiga et al. [9] evaluated the predictors of all-cause mortality over 12 months in a sample of 186 community-based nonagenarians. They observed older age, history of heart failure, and poor nutritional status to be significantly associated with mortality. Disability and cognitive impairment were found to be significant risk factors for mortality after 15 months, in 2249 participants aged 93 years, included in the Danish 1905 – Cohort Survey [10]. In the Vitality 90+ study conducted on 1370 adults aged 90 years physical, psychological, and social components were assumed as indicators of successful aging and found to be significantly associated with higher survival after 4 and 7 years. In addition, the authors found the association to be stronger in women compared to men [11]. The Leiden 85-plus study comprised 599 participants aged 85 years at baseline and followed over 12 years. Several predictors of survival were investigated by the authors that found gait speed [12], Instrumental Activities of Daily Living [12], handgrip strength [13], anemia [14], and changes in the estimated Glomerular Filtration Rate [15] to be significantly associated to survival in the studied cohort. However, previous studies on this topic are still limited in number and findings have been inconsistent. This does not reflect a lack of interest in this topic, but difficulties in reaching the target population and collecting information, mostly due to the high rate of cognitive and functional impairment and frequent institutionalization of participants. As more data on risk factors for mortality in nonagenarians are needed, this study aims to further elucidate predictors of one-year mortality using the data collected in a large sample of nonagenarians that underwent a comprehensive assessment including sociodemographic, cognitive and functional data, lifestyle, and medical history. Data presented in this work were attained in the framework of the Mugello Study, a longitudinal clinic-epidemiological study on people aged 90 years and more living in the Mugello area (Florence, Italy), conducted between 2009 and 2011.

## Methods

### Study design

A detailed description of the Mugello Study protocol has previously been published [16]. The Mugello study is a population-based survey performed between 2009 and 2011 including all persons living in the Mugello area, aged 90 years and over, living both at home or in a residential care facility. Subjects were approached irrespective of their health or cognitive status. Among 1052 eligible subjects 178 were deceased before being interviewed and 148 were not found. The participation rate among contacted subjects was 69% [16]. Information on sociodemographic data, cognitive and functional status, lifestyle, medical history, and drug use was collected either at participants’ homes or in retirement clinics through questionnaires administered by trained physicians, who also performed a though clinical and functional assessment. In the cases where the participant was cognitively impaired, or otherwise not able to be interviewed, a proxy was asked to complete the survey. The Mugello Study protocol complied with the principles of the Declaration of Helsinki on clinical research involving humans and was approved by the Ethical Committee of the Fondazione Don Carlo Gnocchi. Participants, or their legal representative, signed the informed consent form to participate in the study.

### Variables of interest

Information about age, marital status, and smoking habits were collected through a questionnaire.

According to the aim of the study, and considering that centenarians represent a special population of elderly who differ in their comorbidity trends before death compared to nonagenarians [17–20], participants aged 100 or more years at baseline were excluded from the analysis. Drug consumption, presence of depression, and presence of comorbidities were ascertained through clinical evaluations performed via physical examination and interviews with participants and/or caregivers and by reviewing medical records and chronic drug prescriptions. Cognitive function was evaluated using the Mini-Mental State Examination (MMSE) [21]. The total score is comprised between 0 and 30, with lower scores indicating a worse cognitive status. The total score is usually adjusted according to age and education although score correction is not available for people aged 90 years and more. As a consequence, common cut-offs used for this scale were deemed as not adequate to stratify the sample group. Participants were thus stratified according to raw score quartiles, as proposed in previous works conducted on the same cohort [22]. The Short Physical Performance Battery (SPPB) was administered to evaluate walking speed, standing balance, and the ability to stand up from a chair. A score lower than 10 indicates mobility limitation and frailty [23].The level of autonomy was measured using the Basic Activities of Daily Living (BADL) namely eating, bathing, dressing, toileting, and transferring [24]. Continence was not considered, following the recommendation in literature, as it has been suggested that continence should be regarded as a separate dimension and difficulties in bladder and/or bowel control should be considered as an impairment rather than a disability [25]. Participants were considered “independent” if they were able to perform all the considered activities without help, “moderately dependent” if they needed help in one or two activities, and “completely dependent” if they needed assistance in more than two activities [10]. The level of regular physical activity performed over the previous 12 months was assessed through a questionnaire modeled on the Harvard Alumni Questionnaire [26] and specifically adapted for Italian people [27] that assigns a score ranging from 0 (sedentary) to 4 (intense physical activity several times a week). According to the score obtained, participants were divided into two groups, sedentary (no physical activity) versus active (light to intense physical activity). Food consumption was recorded using a specifically designed questionnaire, Mediterranean Diet Score (MedDietScore) scoring from 0 to 55, with higher scores indicating better adherence to the Mediterranean diet [28]. Basing on previous research in nonagenarians [9, 10, 29–33], all collected measures were tested as predictors of mortality.

Finally, information about the survival of each participant 1 year after the date of the interview was obtained from the municipal registers. Follow up data were collected between 2010 and 2012.

### Statistical analysis

Data analysis was performed using SPSS software version 20.0 (SPSS Inc., Chicago, IL, USA). Normally and not normally distributed continuous variables were reported as means ± standard deviations (SD) or median and interquartile range (IQR), respectively. The normality of data distribution was assessed using the Shapiro Wilk test. Dichotomous and categorical data were reported as numbers and percentages. Cox proportional-hazard regression analysis was used to identify possible predictors of mortality within 12 months. Survival time was calculated from examination to death or the end of the follow-up if the participant survived. In the latter case, the survival time was regarded as censored. In a first analysis, proportional hazard models adjusted only by age and sex were created including each variable of interest as an independent variable to investigate its association with mortality. Proportional hazard assumption was tested using Cox regression with a time-dependent covariate analysis. The proportional hazard assumption was deemed as satisfied as the factor * time interaction was non-significant (*p* > 0.05) for all variables tested. Subsequently, all variables significantly associated (*p* < 0.05) to a higher mortality rate, after being adjusted for age and sex, were included in the final analysis. Multicollinearity was tested by Variance inflation Factor (VIF) for all variables included in the final model and showed a VIF < 2 which was deemed as acceptable. Kaplan-Meier curves were then created for time-event analysis and log-rank tests were performed for those variables found to be significantly associated with mortality. In all mentioned analyses, the level of significance was set at a *p*-value< 0.05.

## Results

**Fig. 1:**
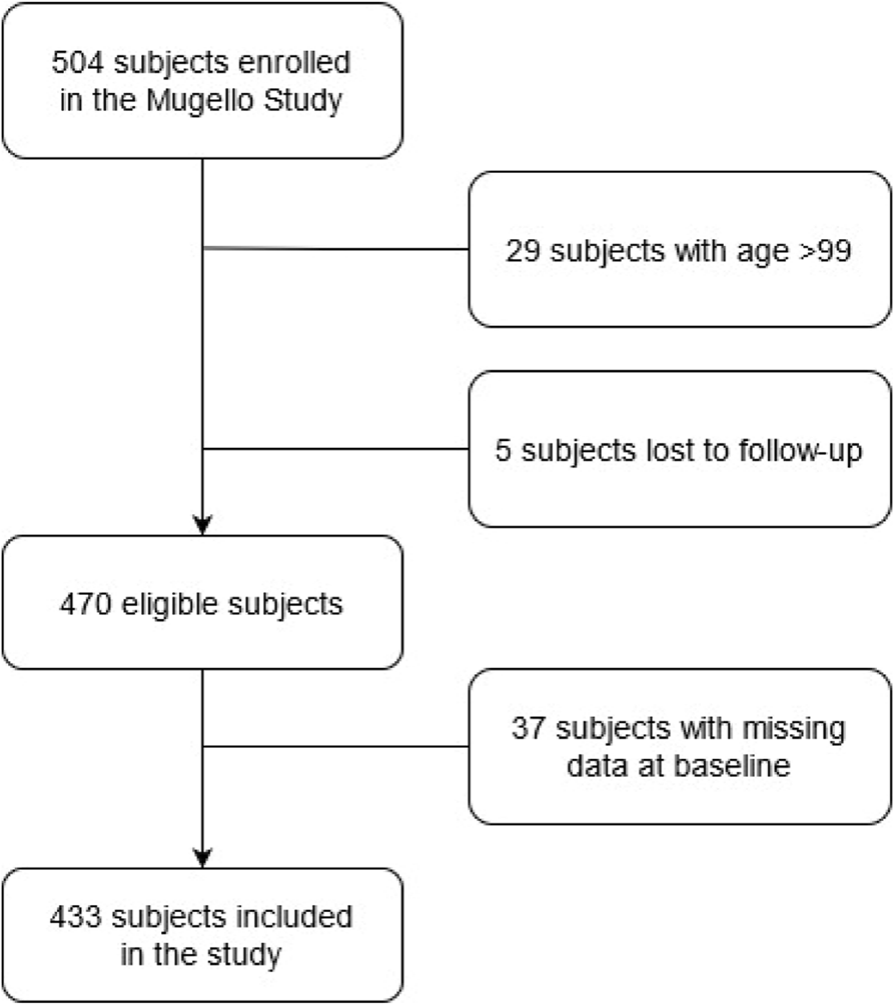
Patients’ flow

Among the 504 subjects enrolled in the Mugello Study and interviewed at baseline, 475 participants had an age comprised between 90 and 99 years. Information about survival at 12 months from the interview was retrieved for 470 subjects. Among these, four hundred and thirty-three individuals had completed data at baseline and were thus included in this study. The patients’ flow of the present study is depicted in Fig. 1.

Individuals excluded from the study due to incomplete data at the baseline were similar to those included in the study for age, sex, sociodemographic characteristics, cognitive and functional status, lifestyle, medical history, and drug use. Compared to subjects included in the study, those excluded reported a lower prevalence of peripheral vascular disease (3% vs 19%, *p* = 0.035) and a higher percentage of institutionalization (24% vs 10%, *p* = 0.013). Characteristics of included and excluded subjects are summarized in Table 1S (Supplementary material).

The study sample was composed of 314 women (72.5%) and 119 men (27.5%) with a median age of 92 years for women and 91 years for men (range 90-99 years). The mortality rate during the follow-up period was 20.3% (88 deaths), with no significant difference between women and men (22.0 and 16.0%, respectively; *p* = 0.165). For those subjects that died during the follow-up, the mean survival time from interview to death was 185 days ranging from 2 to 361 days. The characteristics of the study sample and the two sub-groups, individuals deceased within 12 months and individuals alive at follow-up, are summarized in Table 1.

**Table 1:** Characteristics of the study group

	Total sample (*N* = 433)	Alive (*N* = 345)	Deceased (*N* = 88)
Age (years)	92 [5]	92 [4]	94 [5]
Sex (F)	314 (72.5%)	245 (71.0%)	69 (78.4%)
Time from interview to death (days)	185 ± 101		
Marital status			
Single	19 (4.4%)	14 (4.1%)	5 (5.7%)
Married	82 (18.9%)	70 (20.3%)	12 (13.6%)
Widow/widower	332 (76.7%)	261 (75.7%)	71 (80.7%)
Institutionalized (Y)	43 (9.9%)	31 (9.0%)	12 (13.6%)
Education			
None	63 (14.5%)	52 (15.1%)	11 (12.5%)
Primary school	320 (73.9%)	249 (72.2%)	71 (80.7%)
Secondary school	23 (5.3%)	21 86.1%)	2 (2.3%)
High school or higher	27 (6.2%)	23 (6.7%)	4 (4.5%)
MMSE			
< 14	107 (24.7%)	67 (19.4%)	40 (45.5%)
14-21	97 (22.4%)	77 (22.3%)	20 (22.7%)
22-26	106 (24.5%)	87 (25.2%)	19 (21.6%)
27-30	123 (28.4%)	114 (33.0%)	9 (10.2%)
SPPB score > 9	25 (5.8%)	23 (6.7%)	2 (2.3%)
Autonomy BADL			
Severely dependent	163 (37.6%)	106 (30.7%)	57 (64.8%)
Moderately dependent	125 (28.9%)	106 (30.7%)	19 (21.6%)
Independent	145 (33.5%)	133 (38.6%)	12 (3.6%)
Physical activity score (Active)	197 (45.5%)	180 (52.2%)	17 (19.3%)
Depression (Y)	31 (7.2%)	23 (6.7%)	8 (9.1%)
Smoking			
Never	300 (69.3%)	236 (68.4%)	64 (72.7%)
Former smoker	121 (27.9%)	100 (29.0%)	21 (23.9%)
Current smoker	12 (2.8%)	9 (2.6%)	3 (3.4%)
Nr comorbidities			
0	55 (12.7%)	40 (11.6%)	15 (17.0%)
1-2	265 (61.2%)	217 (62.9%)	48 (54.5%)
> 2	113 (26.1%)	88 (25.5%)	25 (28.4%)
Cancer (Y)	57 (13.2%)	45 8(13.0%)	12 (13.6%)
Myocardial Infarction (Y)	53 (12.2%)	41 (11.9%)	12 (13.6%)
Congestive heart failure (Y)	93 (21.5%)	70 (20.3%)	23 (26.1%)
Peripheral vascular disease (Y)	80 (18.5%)	66 (19.1%)	14 (15.9%)
Hypertension (Y)	253 (58.4%)	201 (58.3%)	52 (59.1%)
Dyslipidaemia (Y)	48 (11.1%)	39 (11.3%)	9 (10.2%)
Chronic lung disease (Y)	55 (12.7%)	49 (14.2%)	6 (6.8%)
Bedsore (Y)	60 (13.9%)	50 (14.5%)	10 (11.4%)
Diabetes without organ damage (Y)	48 (11.1%)	37 (10.7%)	11 (12.5%)
Diabetes with organ damage (Y)	22 (5.1%)	16 (4.6%)	6 (6.8%)
Kidney dysfunction (Y)	23 (5.3%)	13 (3.8%)	10 (11.4%)
MedDietScore	34 [4]	34 [5]	34 [4]
Nr of drugs	3 [2]	3 [2]	3 [3]

Median [interquartile range]

Count (percentage)

*Y* yes, *MMSE* Mini-Mental State Examination, *SPPB* Short Physical Performance Battery, *BADL* Basic Activities of Daily Living, *MedDietScore* Mediterranean Diet Score

Table 2 shows variables’ association with mortality after adjustment for age and sex. Significantly higher risk of dying within 12 months was observed in individuals with severely impaired cognitive status (HR = 5.011, *p* < 0.001 compared to individuals with MMSE score 27 to 30), higher level of dependence in the BADL (HR = 4.193, *p* < 0.001 compared to independent individuals), and sedentary lifestyle (HR = 3.367, *p* < 0.001). A higher number of drugs assumed (HR = 1.118, *p* = 0.031), was also a significant predictor of mortality. Among evaluated comorbidities, only the presence of kidney dysfunction (HR = 2.609, *p* = 0.004) was found to be significantly associated with increased mortality within 1 year.

**Table 2:** Cox regression analysis adjusted for age and sex of all risk factors for mortality at 12 months

	HR	95%CI Lower	95%CI Upper	*p*-value
Marital status (single = ref) Married	0.492	0.164	1.475	0.205
Widow/widower	0.611	0.245	1.526	0.292
Institutionalized (Y)	1.285	0.693	2.383	0.427
Education (High school or higher = ref) None	1.559	0.822	2.956	0.174
Education (High school or higher = ref) Primary school	0.547	0.121	2.474	0.433
Education (High school or higher = ref) Secondary school	1.175	0.367	3.762	0.786
**MMSE (27-30 = ref)**				
**< 14**	**5.011**	**2.354**	**10.667**	**< 0.001**
**14-21**	**2.716**	**1.228**	**6.007**	**0.014**
**22-26**	**2.562**	**1.158**	**5.672**	**0.020**
SPPB score > 9	0.478	0.116	1.966	0.306
**Autonomy BADL (Independent = ref)** **Severely dependent**	**4.193**	**2.196**	**8.006**	**< 0.001**
**Autonomy BADL (Independent = ref)** **Moderately dependent**	1.808	0.875	3.737	0.110
**Physical activity score (Active = ref)** **Sedentary**	**3.367**	**1.943**	**5.835**	**< 0.001**
Depression (Y)	1.496	0.721	3.101	0.279
Smoking (Never = ref) Former smoker	0.999	0.566	1.762	0.998
Smoking (Never = ref) Current smoker	1.480	0.456	4.801	0.514
MedDietScore	0.959	0.912	1.008	0.097
Nr comorbidities (0 = ref) > 2	0.885	0.464	1.690	0.712
Nr comorbidities (0 = ref) 1-2	0.625	0.350	1.117	0.113
**Nr of drugs**	**1.118**	**1.010**	**1.237**	**0.031**
Cancer (Y)	1.120	0.608	2.062	0.717
Myocardial Infarction (Y)	1.198	0.648	2.214	0.564
Congestive heart failure (Y)	1.341	0.834	2.159	0.226
Peripheral vascular disease (Y)	0.927	0.521	1.651	0.798
Hypertension (Y)	1.090	0.706	1.683	0.699
Dyslipidaemia (Y)	1.094	0.542	2.208	0.802
Chronic lung disease (Y)	0.548	0.237	1.267	0.160
Bedsore (Y)	0.787	0.407	1.523	0.478
Diabetes with organ damage (Y)	1.158	0.615	2.180	0.649
Diabetes without organ damage (Y)	1.310	0.570	3.009	0.525
**Kidney dysfunction (Y)**	**2.609**	**1.348**	**5.047**	**0.004**

*Y* yes, *HR* Hazard Risk, *CI* Confidence Interval, *MMSE* Mini-Mental State Examination, *SPPB* Short Physical Performance Battery, *BADL* Basic Activities of Daily Living, *MedDietScore* Mediterranean Diet Score

When all variables were included in the analysis, together with age and sex regarded as a priori confounders, only the presence of older age (HR = 1.079, *p* = 0.048), lower cognitive function (HR = 2.859, *p* = 0.015), sedentary life (HR = 2.030, *p* = 0.026), and kidney dysfunction (HR = 2.322, *p* = 0.018) remained significantly associated with a higher risk of mortality (Table 3).

**Table 3:** Cox regression analysis. Factors significantly associated with mortality at 12 months

	HR	95%CI Lower	95%CI Upper	*p*-value
Age	1.079	1.001	1.163	0.048
MMSE (27-30 = ref)				
< 14	2.859	1.229	6.655	0.015
14-21	1.752	0.754	4.070	0.192
22-26	1.969	0.871	4.451	0.104
Physical activity score (Active = ref)				
Sedentary	2.030	1.089	3.787	0.026
Kidney dysfunction	2.322	1.156	4.665	0.018

*HR* Hazard Risk, *CI* Confidence Interval, *MMSE* Mini-Mental State Examination

Variables not significantly associated with mortality (*p* > 0.05) included in the Model: sex, autonomy in Basic Activities of Daily Living, and number of drugs

**Fig. 2:**
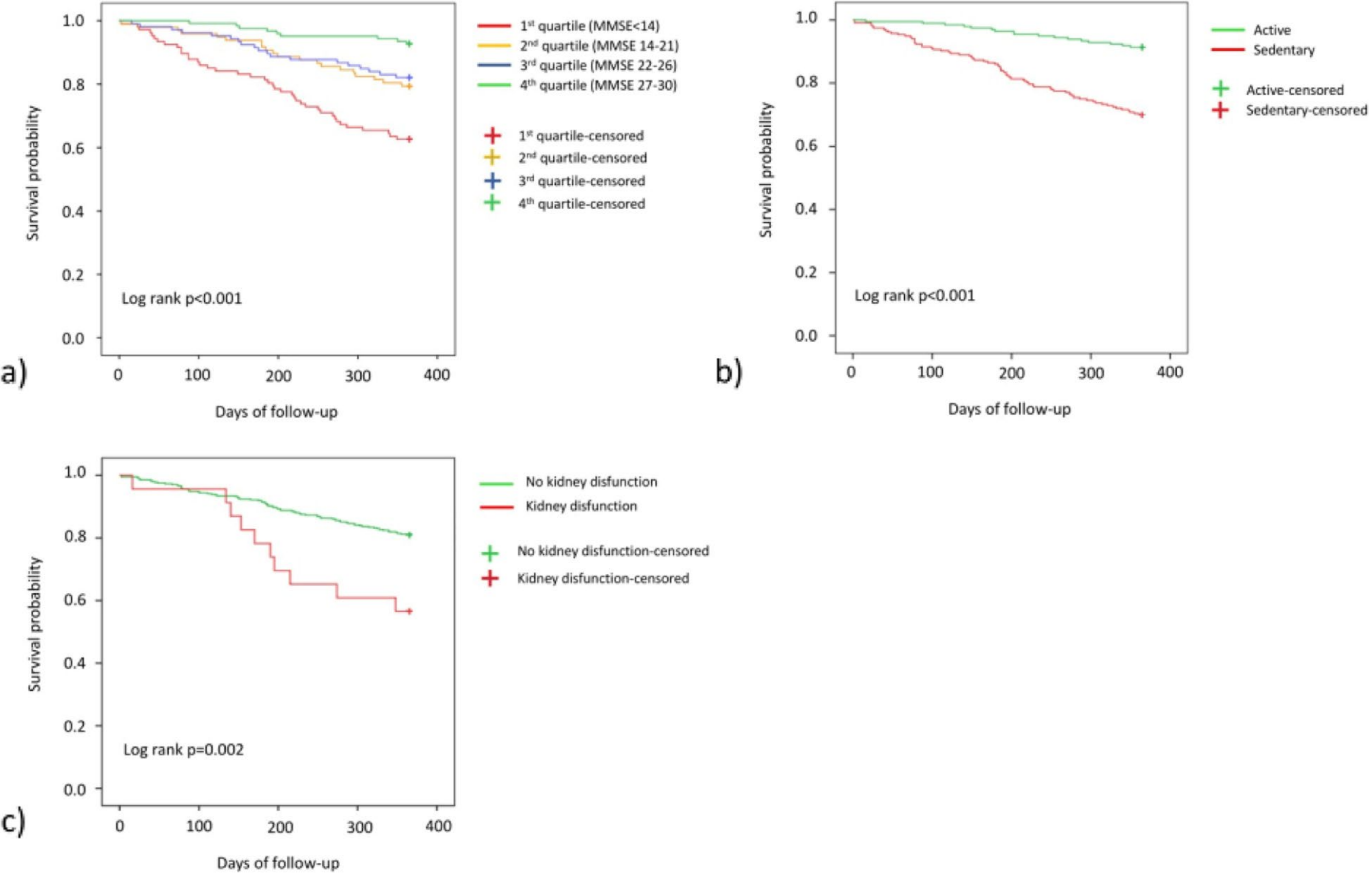
Kaplan-Meier survival curve based on: **a** MMSE score at baseline, **b** reported baseline physical activity, **c** the presence of kidney dysfunction detected at baseline

Kaplan-Meier survival curves at 1 year based on the MMSE score are presented in Fig. 2a. These curves show that belonging to the lower MMSE quartile (score < 14) was associated with the lowest probability of survival within 12 months. The probability increases for increased MMSE values (from second to the fourth quartile, corresponding to a preserved cognitive status). Similarly, the association between the reduced probability of survival and the presence of sedentary life and kidney dysfunction is illustrated through the Kaplan-Meier curves in Fig. 2b and c, respectively.

## Discussion

In the cohort of nonagenarians included in this study, a one-year mortality rate of about 20.3% was observed. The average mortality rate for Italian people aged 92, which coincides with the median age of the sample group, is reported to be 20% (data from the Human Mortality Database [34], concerning the 2010-2012 period). Mortality observed in this cohort seems thus to be in line with the national average rate. This value was slightly higher than what was observed in the NonaSantfeliu Study, where a mortality rate of 19.3% was recorded after 12 months [9], but in line with the one observed in the Danish 1905 study (25.7% mortality after 15 months) [10]. No significant difference in the percentage of non-survivors was recorded between men and women. It has been observed that the gender gap in life expectancy decreases at older ages [35]. Nevertheless, looking at the literature [36] and the Italian national statistics a difference in life expectancy between women and men seems to persists even in the oldest old (national average mortality rate for 92 years old subjects in the 2010-2012 period was 19% for women and 24% for men [34]) even if not detected in this cohort. The percentage of women in the sample (72.5%) was slightly lower than observed in other studies (74% Danish 1905 – Cohort Survey [10], 77% NonaSantfeliu Study [9], 80% Vitality 90+ Study [29]). The percentage of institutionalized subjects in the sample (10%) was considerably lower than reported in previous studies (19% NonaSantfeliu Study [9], 35.2% Vitality 90+ Study [29]). Similarly, the percentage of subjects with more than two comorbidities (26.1%) was markedly lower compared to literature (35% Danish 1905 – Cohort Survey [10], 40% Vitality 90+ Study [29]).

In line with results from previous studies, increasing age [9] and cognitive impairment [9, 21, 29] were found to be associated with mortality at 12 months. The association between impaired cognitive function and mortality is coherent with results found in younger [37] and older populations [38]. However, whether cognitive impairment is merely a marker of medical disease severity or an independent predictor of poor health outcomes and mortality is still unclear [37]. So far, several theories have been formulated in an attempt to explain the association between cognitive decline and mortality. Cognitive decline has been claimed as an indicator of primary brain damage [39] or, more in general, it has been regarded as an indicator of the human organism’s total functioning. According to this latest theory, regardless of the cause of impaired general functioning, similar impairment in cognitive function will occur [40]. An alternative explanation is a decreased ability of cognitively impaired people to cope with everyday activities, including those related to health care [27], as adhering to medication intake, recognizing and reporting worrisome signs and symptoms of medical conditions, being physically active and eating healthy [24]. Moreover, aging of the central nervous system might be expected to reduce adaptive capacity, thereby increasing susceptibility to death from a variety of causes [41]. Another theory proposes cognitive impairment to exacerbate chronic medical illness. The study from Gale et al. addressed this latest hypothesis and reported a significant association between cognitive impairment and death from ischemic stroke in 921 subjects aged 65 years and over [42]. However, following studies on larger samples (7482 individuals, age ≥ 65 years), did not find cognitive impairment to worsen chronic medical illness and found it to be significantly associated with mortality, independently of the number of comorbidities [37].

Whether there is a causative component of cognitive impairment on mortality deserves further investigations. This is especially true for the oldest old population as the pathway by which cognitive impairment leads to poor health outcomes may vary over the life span.

In this study group sedentary lifestyle was also found to be associated with reduced survival. Physical inactivity is a well-recognized risk factor for coronary heart and cerebrovascular diseases, diabetes, hypertension, cancer, and cognitive impairment [43] and there is a large body of literature focusing on the cognitive and physical benefits of exercise training in older adults (age ≥ 60) [44]. More interestingly, even among people aged 80 and over, physical activity seems to be associated with better health outcomes. Findings from Stressman and colleagues suggest that being physically active up to age 85 delays functional loss and improves survival [45]. In addition, they observed physical activity to be beneficial also for those becoming active in advanced age. In the same population, physical inactivity was found to be associated with a great risk of stroke, independently from medical comorbidities [46]. Data from this study corroborate those findings even in the population segment aged 90-99. In addition, although a linear relationship exists between the level of physical activity and health status, results from this cohort suggest, coherently with findings in people aged 85 and over [47], that even a light physical activity may reduce significantly the risk of mortality. Although it cannot be excluded the existence of reverse causality, with physical inactivity being a consequence of accumulated disability from medical comorbidities and frailty, maintaining an active lifestyle may delay the onset of this spiral of decline [45]. Results from this study suggest that physical activity should not be regarded as a central goal only in old adults, but may be an important component of primary prevention also in the oldest old population.

Finally, in this study sample, the presence of kidney dysfunction was significantly and independently associated with the risk of dying. A probable explanation of the association between kidney dysfunction and mortality rate is that poor renal function may be associated with a greater risk for heart disease [48]. In fact, in older adults, kidney function decline was found to be associated with a higher prevalence and increased severity of cardiovascular risk factors such as hypertension and dyslipidemia [49] and it has been suggested to be a marker of atherosclerosis disease progression [48]. In addition, it has been reported to negatively affect cognitive and physical function and to be responsible for decreased appetite, loss of lean body mass, and frailty [50–52]. The results from this study seem to agree with previous research regarding the importance of renal function on survival at older age. Data from the Leiden Longevity Study showed a better renal function in subjects with a propensity for longevity, compared to environmentally matched controls. This association was found only in men and it was more significant in those with hypertension or cardiovascular disease [53]. Similarly, a recent work from Haberle and colleagues investigated the association between kidney function and survival to age 90 [54]. They found both low mean value and a reduction in estimated glomerular filtration rate (eGFR) to be significantly associated with a lower likelihood of reaching age 90. However, they observed a moderate attenuation of the effect of eGFR level on survival with age which was not confirmed by our results. In this study, the relationship between reduced renal function and the exacerbation of cardiovascular risk factors could have been elucidated by knowing the participants’ cause of death. As retrieving this information was not possible in this sample, future research is needed to confirm this hypothesis.

In contrast with findings from previous studies [9] heart failure and poor nutritional status were not significantly related to survival after 1 year. Compared to the sample observed by Formiga and colleagues, in this study group, a lower prevalence of heart failure was observed in non-survivors (26.1% vs 58.3%), which may partially explain the different results obtained. The prevalence seen in this sample seems to be coherent with a previous work reporting heart failure history in a small sample of nonagenarians (prevalence 23%, [55]), and even lower prevalence is reported in larger samples (59,423 subjects aged 90 and over, prevalence 11.2% [56]). Regarding adherence to the Mediterranean diet, scores observed were comparable to those recorded in a small sample of nonagenarians (*N* = 19, MedDietScore = 34 ± 3) living in a Mediterranean country (Greece) [57]. In addition, the lack of association between mortality and dietary patterns observed in this cohort corroborates the results from Lasheras et al. [58] that found the Mediterranean diet to be significantly associated with reduced mortality in people aged less than 80 years, but not in people aged 80 years and over. The discrepancies with the results obtained by Formiga et al. [9] can be related to the different assessments performed. In fact, in our study, the evaluation focused on the general profile of food and nutrient consumption, while the work of Formiga and colleagues considered the nutritional status (including body mass index, appetite, weight loss, mobility, current illness, and neuropsychological problems) which is affected not only by the quality and quantity of food consumed but also by the general health of the subject.

Nonagenarians included in this study were less independent compared to previously investigated cohorts [10] (percentage of severely dependent individuals 37.6% vs 21%) and decreased ability in performing BADL was found to be related to reduced survival, but the effect did not remain significant when the analysis was adjusted for all risk factors. Consistently with findings from The Danish 1905 study [10] smoking was not found to be associated with reduced survival, although it is a widely recognized risk factor for mortality [59]. The extent to which genetic and environmental characteristics may have protected nonagenarians against tobacco-related diseases, as suggested by Nybo and colleagues [10], remains an open question as well as how much these individuals would have benefit in terms of quality and length of survival from never having smoked. Similarly, the number of comorbidities did not seem to affect survival at 1 year. A possible explanation for the lack of association between the number of comorbidities and mortality has been suggested by Nybo et al. [10]. They hypothesized that age-related decline in the function of the organs and acute infections have a primary role in nonagenarians’ mortality and that this is not reflected by the number of diseases. However, different conclusions were drawn by Halonen and colleagues that found multimorbidity to be significantly associated with mortality [29]. As discrepancies in findings among studies may be due to different comorbidities inclusion and assessment, further investigations are needed to explore the relationship between multimorbidity and mortality in this cohort. Summarizing, nonagenarians may differ from centenarians in their comorbidity trends before death and factors that play a role in mortality [17–20], and, in the same way, may differ from younger age groups in risk factors [33] and frailty [60], suggesting the peculiarity of this category of elderly and the need to study that separately in comparison with younger and older categories.

A limitation of this study that deserves to be acknowledged concerns the self-reported nature of clinical diagnoses although this limitation was partially overcome by a through clinical anamnesis and examination, by reviewing the medical records of the participants and by double-checking the information provided with their proxies. Moreover, missing information did not allow to clarify whether unfavorable conditions (such as severe kidney failure) were the primary cause of death. Despite these limitations, this work confirms some of the findings obtained in previous studies and gives further insight into survival in this rapidly increasing slice of the population, approaching a lifespan of a century.

## Conclusions

Data from the Mugello study support the hypothesis that mortality in nonagenarians is not a stochastic process, and that the risk factors associated with mortality in the oldest old may be somewhat different from those already identified for the old population altogether. In summary, findings from this study suggest that older age, reduced cognitive function, sedentary lifestyle, and the presence of kidney dysfunction are associated with mortality in nonagenarians.

## Supplementary Information

**Additional file 1.**

## Data Availability

The datasets generated and/or analysed during the current study are not publicly available due on our policy statement of sharing clinical data only on request but are available from the corresponding author on reasonable request.

## Abbreviations

BADL: Basic Activities of Daily Living
SPPB: Short Physical Performance Battery
MMSE: Mini-Mental State Examination
MedDietScore: Mediterranean Diet Score
SD: Standard deviation
IQR: Interquartile range
HR: Hazard risk
CI: Confidence Interval
eGFR: Estimated glomerular filtration rate
