# Supplementary material for: 12-month survival in nonagenarians inside the Mugello study: on the way to live a century
Source: BMC Geriatr. 2022 Mar 12;22:194. doi: 10.1186/s12877-022-02908-9 (PMC8918304; doi:10.1186/s12877-022-02908-9)
Supplement: Supplementary file 1 — Additional file 1. [file 12877_2022_2908_MOESM1_ESM.docx]

Supplementary Material

Table 1S Characteristics of subjects excluded in the study (subjects with incomplete data) and included (subjects with complete data).

|  | N | Subjects with incomplete data | N | Subjects with complete data | p-value |
| --- | --- | --- | --- | --- | --- |
| Age (years) | 37 | 92 [5] | 433 | 92 [5] | 0.067 |
| Sex (F) | 37 | 26 (70.3%) | 433 | 314 (72.5%) | 0.769 |
| Deceased | 37 | 9 (31%) | 433 | 88 (20.3%) | 0.170 |
| Time from interview to death (days) | 37 | 220±106 | 433 | 185±101 | 0.316 |
| Marital status | 37 |  | 433 |  | 0.565 |
| Single |  | 3 (8.1%) |  | 19 (4.4%) |  |
| Married |  | 7 (18.9%) |  | 82 (18.9%) |  |
| Widow/widower |  | 27 (73.0%) |  | 332 (76.7%) |  |
| **Institutionalized (Y)** | **37** | **9 (24.3%)** | **433** | **43 (9.9%)*** | **0.013** |
| Education | 28 |  | 433 |  | 0.623 |
| None |  | 4 (14.3%) |  | 63 (14.5%) |  |
| Primary school |  | 19 (67.9%) |  | 320 (73.9%) |  |
| Secondary school |  | 2 (7.1%) |  | 23 (5.3%) |  |
| High school or higher |  | 3 (10.7%) |  | 27 (6.2%) |  |
| MMSE | 37 |  | 433 |  | 0.060 |
| <14 |  | 16 (43.2%) |  | 107 (24.7%) |  |
| 14-21 |  | 8 (21.6%) |  | 97 (22.4%) |  |
| 22-26 |  | 4 (10.8%) |  | 106 (24.5%) |  |
| 27-30 |  | 9 (24.3%) |  | 123 (28.4%) |  |
| SPPB score >9 | 37 | 2 (5.4%) | 433 | 25 (5.8%) | 0.926 |
| Autonomy BADL | 28 |  | 433 |  | 0.419 |
| Severely dependent |  | 14 (50.0%) |  | 163 (37.6%) |  |
| Moderately dependent |  | 6 (21.4%) |  | 124 (28.6%) |  |
| Independent |  | 8 (28.6%) |  | 145 (33.5%) |  |
| Physical activity score (Active) | 37 | 17 (45.9%) | 433 | 197 (45.5%) | 0.913 |
| Depression (Y) | 37 | 4 (15.4%) | 433 | 31 (7.2%) | 0.343 |
| Smoking | 32 |  | 433 |  | 0.633 |
| Never |  | 23 (71.9%) |  | 300 (69.3%) |  |
| Former smoker |  | 9 (28.1%) |  | 121 (27.9%) |  |
| Current smoker |  | 0 (0.0%) |  | 12 (2.8%) |  |
| Nr comorbidities | 30 |  | 433 |  | 0.057 |
| 0 |  | 8 (26.7%) |  | 55 (12.7%) |  |
| 1-2 |  | 18 (60.0%) |  | 265 (61.2%) |  |
| >2 |  | 4 (13.3%) |  | 113 (26.1%) |  |
| Cancer (Y) | 30 | 2 (7.7%) | 433 | 57 (13.2%) | 0.405 |
| Myocardial Infarction (Y) | 30 | 6 (20.0%) | 433 | 53 (12.2%) | 0.252 |
| Congestive heart failure (Y) | 30 | 6 (20.0%) | 433 | 93 (21.5%) | 0.849 |
| **Peripheral vascular disease (Y)** | **30** | **1 (3.3%)** | **433** | **80 (18.5%)** | **0.035** |
| Hypertension (Y) | 30 | 14 (46.7%) | 433 | 253 (58.4%) | 0.207 |
| Dyslipidaemia (Y) | 30 | 1 (3.3%) | 433 | 48 (11.1%) | 0.350 |
| Chronic lung disease (Y) | 30 | 3 (10.0%) | 433 | 55 (12.7%) | 0.665 |
| Bedsore (Y) | 30 | 3 (10.0%) | 433 | 60 (13.9%) | 0.783 |
| Diabetes without organ damage (Y) | 30 | 0 (0.0%) | 433 | 48 (11.1%) | 0.060 |
| Diabetes with organ damage (Y) | 30 | 0 (0.0%) | 433 | 22 (5.1%) | 0.385 |
| Kidney dysfunction (Y) | 30 | 1 (3.3%) | 433 | 23 (5.3%) | 0.636 |
| MedDietScore | 34 | 34 [6] | 433 | 34 [4] | 0.812 |
| Nr of drugs | 35 | 3 [2] | 433 | 3 [2] | 0.910 |
| Y=yes  Median [interquartile range]  Count (percentage)  MMSE = Mini-Mental State Examination; SPPB = Short Physical Performance Battery; BADL = Basic Activities of Daily Living; MedDietScore: Mediterranean Diet Score | | | | | |
